# Effects of Bi Addition on the Microstructure and Mechanical Properties of Nanocrystalline Ag Coatings

**DOI:** 10.3390/ma10080932

**Published:** 2017-08-10

**Authors:** Yuxin Wang, Guang Cheng, See Leng Tay, Yunxia Guo, Xin Sun, Wei Gao

**Affiliations:** 1 School of Materials Science and Engineering, Jiangsu University of Science and Technology, Zhenjiang 212003, Jiangsu, China; ywan943@163.com (Y.W.); yunxiajust@163.com (Y.G.); 2 Department of Chemical & Materials Engineering, the University of Auckland, PB 92019, Auckland 1142, New Zealand; seeleng.tay@cirrusmaterials.com (S.L.T.); w.gao@auckland.ac.nz (W.G.); 3 Physical and Computational Sciences Directorate, Pacific Northwest National Laboratory, P.O. Box 999, Richland, WA 99352, USA; 4 Energy and Transportation Science Division, Oak Ridge National Laboratory, 1 Bethel Valley Road, Oak Ridge, TN 37830, USA; sunx1@ornl.gov

**Keywords:** electrodeposited nanocrystalline Ag, Bi addition, microstructure, nanoindentation, mechanical properties, electrical conductivity

## Abstract

In this study we investigated the effects of Bi addition on the microstructure and mechanical properties of an electrodeposited nanocrystalline Ag coating. Microstructural features were investigated with transmission electron microscopy (TEM). The results indicate that the addition of Bi introduced nanometer-scale Ag-Bi solid solution particles and more internal defects to the initial Ag microstructures. The anisotropic elastic-plastic properties of the Ag nanocrystalline coating with and without Bi addition were examined with nanoindentation experiments in conjunction with the recently-developed inverse method. The results indicate that the as-deposited nanocrystalline Ag coating contained high mechanical anisotropy. With the addition of 1 atomic percent (at%) Bi, the anisotropy within Ag-Bi coating was very small, and yield strength of the nanocrystalline Ag-Bi alloy in both longitudinal and transverse directions were improved by over 100% compared to that of Ag. On the other hand, the strain-hardening exponent of Ag-Bi was reduced to 0.055 from the original 0.16 of the Ag coating. Furthermore, the addition of Bi only slightly increased the electrical resistivity of the Ag-Bi coating in comparison to Ag. Results of our study indicate that Bi addition is a promising method for improving the mechanical and physical performances of Ag coating for electrical contacts.

## 1. Introduction

Silver (Ag) has been widely applied in the semiconductor industry because of its excellent thermal and electrical conductivity [1,2,3]. However, the wear resistance of Ag is not as desirable as those of nickel (Ni) or copper (Cu) [3,4,5] due to the fact that bulk Ag or Ag coatings are soft, with low hardness. Various methods were attempted to improve the mechanical performance of Ag coatings, such as introducing hard nanoparticles to form Ag-based composites [6,7,8], creating new Ag-based alloys with different crystal structures [1,5,9,10,11,12,13], and reducing the grain size to the hundred nanometer scale [9,14,15,16,17,18]. In addition, different coating/film deposition techniques, i.e., electrodeposition, physical vapor deposition (PVD), chemical vapor deposition (CVD), and magnetron sputtering, have been employed to prepare Ag coatings with improved properties [19]. Among those techniques, electrodeposition has attracted a great deal of research interest since this technique could prepare metallic alloys with unique compositions and novel micro/nanostructures, and, therefore, improve performance significantly [7,14,20].

An ideal electrical contact material should possess high electrical conductivity and high wear resistance/hardness. However, among the various electrodeposited Ag alloy systems, the electrical conductivity and strength of alloys have always been two competing factors. In order to obtain stronger Ag alloys, higher alloying compositions are typically employed which increase electrical resistivity and, therefore, compromise conductivity [21]. Naturally, an Ag alloy system with low solubility could become a good candidate for realizing desired performance. Among different Ag-based alloy systems, the solubility of bismuth in solid-state silver is very low (~1.5 at%), according to the phase diagram [22], as well as the silver-bismuth (Ag-Bi) systems that have been attempted in previous studies with mechanical alloying [11] and electrodeposition [3,23]. The small amount (<1 at%) of Bi addition has the potential for improving the mechanical performances with only a slight decrease of electrical conductivity when the solid solutions are formed. However, so far no study reported any improved mechanical properties of the Ag-Bi alloy systems in comparison with those of pure Ag.

In addition to the alloy system design, the electrodeposition process typically generates columnar or lamellar coating structures, depending on the processing parameters, e.g., pulsed wave forms, current density, temperature, bath composition and constituents, additive type and concentration, and metal ion concentration. Previous studies have shown that coatings prepared by different processing methods have directionally-dependent elastic-plastic properties due to their microstructural-level anisotropy [24,25,26,27]. Nevertheless, considering the thin nature of the coatings (with thickness from ~1 to 100 μm), only small-scale testing and characterization methods, such as free-standing (FS) thin film tensile [28,29,30], micro-cantilever deflection [31,32], nanoindentation [31,33,34,35,36,37], and micro-pillar compression [38,39,40], can be applied to characterize the coating mechanical properties. Some studies have addressed the anisotropic behaviors of coatings [24,25,26,27]; however, very few studies quantified the elastic-plastic anisotropy of thin coatings, i.e., thickness below 10 µm, due to the difficulty of small-scale sample preparation. Meanwhile, an additional question should be clarified: how low of an at% element addition will influence the anisotropy of the alloy coatings, from the perspectives of both microstructural features and mechanical properties? Thus, it is necessary to conduct microstructural and mechanical characterizations in order to quantify the properties of coatings, as well as to illustrate the effects of new alloy element addition on the coating anisotropy. 

In this study, we first characterized the microstructural features of the coatings using scanning electron microscopy (SEM) and X-ray diffraction (XRD). Next, we investigated the microstructures of the Ag and Ag-Bi nanocrystalline using TEM and high-resolution transmission electron microscopy (HRTEM) to characterize the effects of Bi addition on the microstructural features at the nano-scale. Then, we applied the recently-reported nanoindentation-based technique [27] to interrogate the anisotropic elastic-plastic properties of the Ag-Bi coating. The technique was developed by the authors to extract the anisotropic elastic-plastic properties of electrodeposited Ag coating [27] with nanoindentation in conjunction with three-dimensional (3-D) finite element analyses (FEA). Previous studies have revealed that the inverse flow stress from the nanoindentation is consistent with the tensile flow curves measured by free-standing (FS) Ag film [29]. In the current study, we applied the nano-indentation-based technique [27] to the electrodeposited Ag-Bi coating in order to calculate the flow stress of Ag-Bi to compare with previously studied Ag coating, hence, the effects of Bi addition on the mechanical properties of Ag coating were quantified. Finally, we presented a systematic discussion of the mechanical properties and electrical conductivity.

## 2. Materials and Methods

### 2.1. Sample Preparation and Characterization

**Figure 1:**
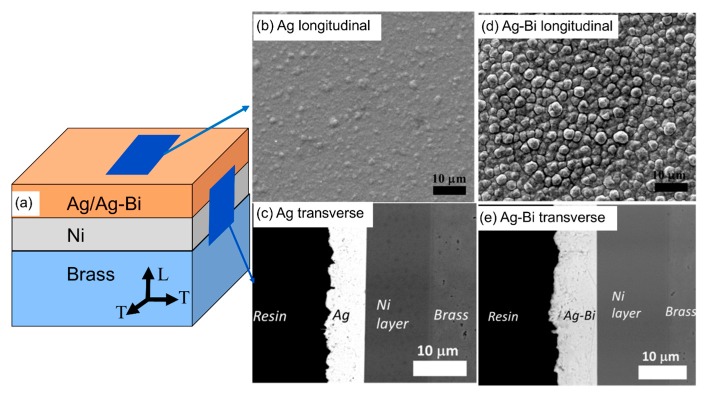
(**a**) The sketch of Ag/Ag-Bi coatings; (**b**) scanning electron microscope (SEM) image of Ag coating on as-deposited surfaces; (**c**) SEM image of Ag coating on the polished surfaces perpendicular to the *T* direction; (**d**) SEM image of Ag-Bi coating on the as-deposited surfaces; and (**e**) SEM image of Ag-Bi coating on the polished surfaces perpendicular to the *T* direction.

Nanocrystalline Ag and Ag-Bi coatings were electrodeposited onto a Ni-coated brass substrate (20 × 20 × 0.6 mm) as illustrated in Figure 1a. The solution composition and operating conditions are listed in Table 1. The detailed description of the deposition process was reported by Tay [14]. The surface morphology and composition of coatings were analyzed using a field emission scanning electron microscope (FESEM) equipped with an energy dispersive spectroscope (EDS) in Figure 1b,d. The surface morphology of the Ag coating is ambiguous compared with that of the Ag-Bi coating due to the low contrast and relatively plain surface. The at% of Bi within the Ag-Bi coating was measured as ~1%. The thickness of Ag-Bi and Ni was ~10 μm and ~7 µm, respectively, as shown in Figure 1c,e. 

**Figure 2:**
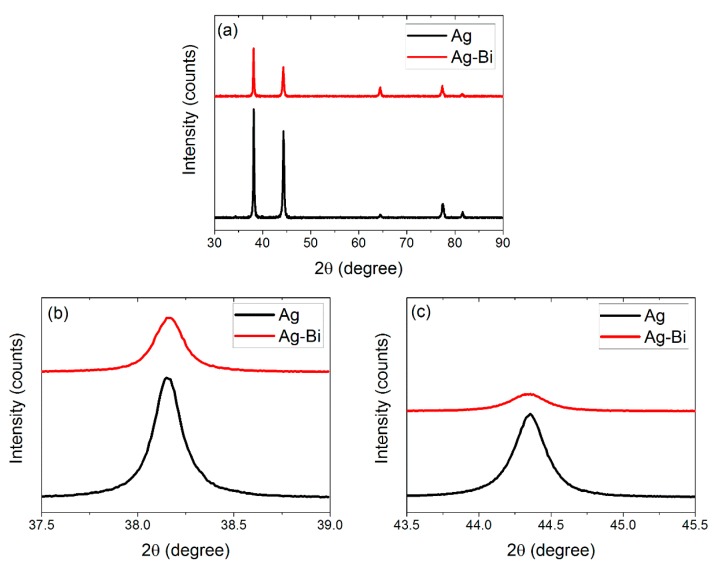
(**a**) XRD pattern of Ag and Ag-Bi coatings; (**b**) the magnified peak of Ag (111); and (**c**) the magnified peak of Ag (200).

The crystal structure was characterized by X-ray diffraction (XRD) with Cu Kα radiation (D2 Phaser Bruker, Karlsruhe, Germany, V = 30 kV, I = 10 mA). Diffraction patterns were recorded in the 2 theta range from 35 to 85° with a scanning step of 0.01°, as shown in Figure 2. 

The electrical resistivity was measured by the four-point probe method after the Ag and Ag-Bi coatings were peeled off from the substrate and were layered on a silicon substrate. An electric current was passed through the two outer probes, and the potential was measured between two inner probes with a Keithley 2602 meter (Cleveland, OH, USA). The electrical resistivity was calculated using a standard method [41]. The peeled-off coatings were thinned using an ion beam milling system and the TEM images were taken by a high-resolution TEM (FEI TECNAI G2 F20, Hillsboro, OR, USA, 200 kV).

### 2.2. Nanoindentation

**Figure 3:**
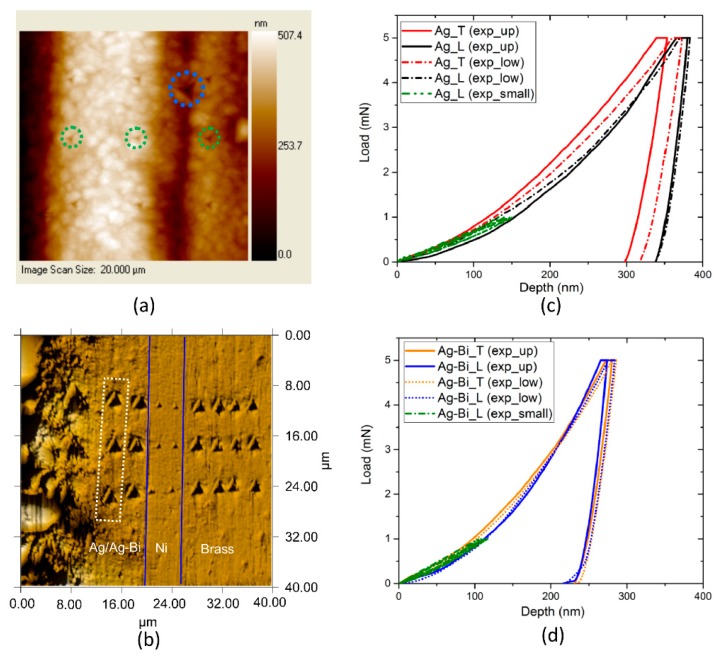
(**a**) Indents (highlighted in circles) in the *L* direction under SPM with contour color bars indicating the surface height (the indents highlighted in green circles denoted an indentation ~100 nm and the indent highlighted in the blue dotted circle denoted an indentation with P_max = 5 mN); (**b**) indents in the *T* direction under SPM (the indents highlighted in the white box are used for inverse calculation); (**c**) The upper bound (solid) and lower bound (dotted) of the P-h curves obtained in the two directions for the Ag coating and the small depth (~150 nm) P-h curves in the *L* direction; and (**d**) the upper bound (solid) and lower bound (dotted) of the P-h curves obtained in the two directions for Ag-Bi coating and the small depth (~120 nm) P-h curves in the *L* direction.

The nanoindentation tests were conducted using a Hysitron TI950 triboindenter (Minneapolis, MN, USA) along the *L* and *T* directions with a diamond Berkovich tip (Minneapolis, MN, USA) as shown in Figure 3a,b. The maximum indentation loading was 5 mN so that the maximum indentation depth was around 300 nm in both directions to avoid the substrate effect. The maximum load was held for 10 s to avoid creep behaviors in both indentation directions. Multiple indents were attempted, and at least three P-h curves in each direction were obtained. The surfaces perpendicular to the *T* directions were polished using a standard method so the influence of surface roughness could be ignored. The typical P-h curves are shown as the solid red curve in Figure 3c,d where the upper bound (the maximum P-h curve) and lower bound (the minimum P-h curve) of each direction are shown. The different indentation responses could be attributed to the microstructural orthotropy of the polycrystalline coating. In our study, the distance of each indent was kept at ~2 μm to reduce the possible residual stress from the surrounding indents [42]. Meanwhile, the indents near the interface were not considered in order to avoid the potential effects of Ni substrate on the indents in the *T* direction.

The surface perpendicular to the *L* direction, i.e., the as-deposited surface, could not be polished to minimize surface roughness due to the very thin nature of the coatings. To reduce the influence of surface roughness on the P-h curves, the indents were observed under scanning probe microscopy (SPM). As shown in Figure 3a, the color contour illustrates the height differences within the 30 × 30 μm observation region. Hence, the P-h curves in the *L* direction would be influenced by the surface roughness if indents were located on uneven surfaces [43]. If significant differences were observed on the loading curvature in the *L* direction, the P-h curves would be rejected. Additionally, a series of low depth (~120 nm) indents in the *L* direction were conducted before deep depth indentation (~300 nm). The P-h curves were obtained with relatively small scatter as the dotted green curves in Figure 3c,d, and these curves were utilized to guide the selection of the back curve (~300 nm) in the *L* direction. It should be noted that the challenges of surface roughness cannot be completely overcome at such small indentation depths. However, we have taken special care to choose the P-h curves with minimal surface roughness influence for the inverse calculations of Ag and Ag-Bi coatings, as the red and orange curves shown in Figure 3c,d, respectively. The averaged P-h curves from all the indents along a single direction were used for the subsequent inverse analyses.

## 3. Results

### 3.1. Microstructure of the Ag and Ag-Bi Coatings

**Figure 4:**
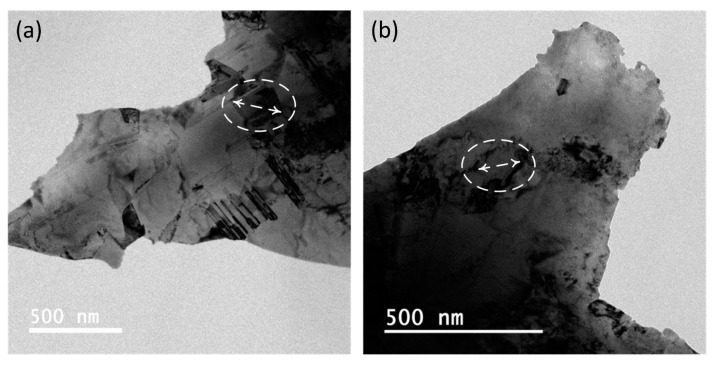
TEM images of the nanocrystalline: (**a**) Ag coating and (**b**) Ag-Bi coating from the plane view with individual grains highlighted in the white dotted box.

The peak intensities of Ag and Ag-Bi coatings are shown in Figure 2; no new phases were generated. However, the Bi element addition would generate the smaller crystallite size [11] and introduce intrinsic lattice distortions to the Ag matrix [44]. The lower-intensity peaks for the Ag-Bi coating were observed. There is a very small shift of peaks to the lower diffraction angle observed in Figure 2b. Then, the microstructures of the Ag and Ag-Bi electrodeposited coatings were characterized using TEM and HRTEM. Equiaxed fine-grains were observed in the plane view TEM images, as shown in Figure 4a. The grain sizes are smaller than 200 nm including nano-sized twins of different thickness. Equiaxed fine-grains of smaller sizes are shown in Figure 4b for the Ag-Bi coating. However, no nano-sized twins were observed in the Ag-Bi coating.

**Figure 5:**
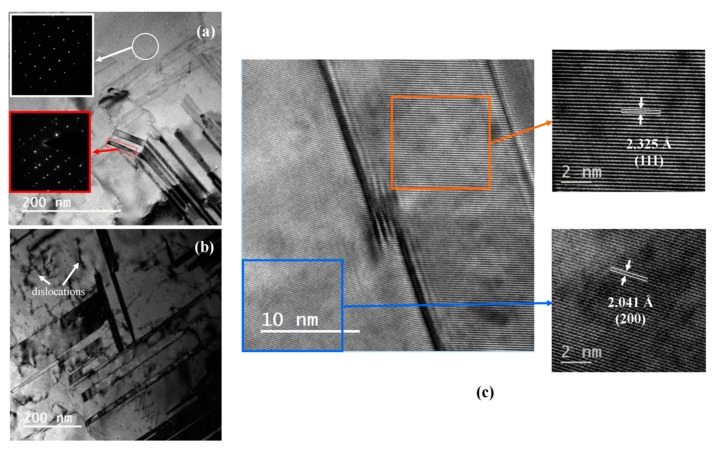
(**a**) The bright field TEM image from the plane view for Ag with selected area diffractions (SAD) patterns to illustrate the FCC structures ([110]) within the white area and nanocrystalline twining within the red area; (**b**) the TEM bright field image of nanocrystalline Ag with dislocations; and (**c**) the HRTEM images with areas in and out of the nanocrystalline twining.

**Figure 6:**
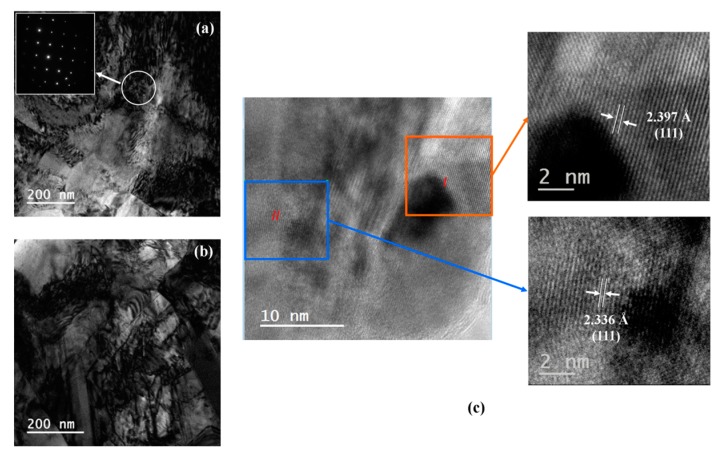
(**a**) The bright field TEM image from the plane view for Ag-Bi with selected area diffractions (SAD) patterns to illustrate a FCC structures ([001]) within highlighted area; (**b**) the TEM bright field image of Ag-Bi alloy with plenty of defects; and (**c**) the HRTEM images with areas in and out of the black Ag-Bi solid solutions.

Further detailed microstructures of these two coatings are shown in Figure 5 and Figure 6. The nano-sized twins were observed within the Ag coating as shown in Figure 5a,b. The orientations of these nano-sized twins are random. The selected area diffraction (SAD) in the Figure 5a pattern from the red dotted region demonstrates a twinning pattern, and the width of these nano-sized twins could be as small as 20 nm. In addition, few dislocations were observed with the Ag grains and are highlighted in Figure 5a. A HRTEM image, as shown in Figure 5c, was taken to calculate the lattice parameters in and out of the twinning grains as two highlighted regions in the orange box and blue box, respectively. The purposes of calculating the lattice parameters were to analyze the possible reasons for generating nano-sized twins in Ag [45] and to verify the existence of Bi in the Ag-Bi coating [11]. The distance between atoms was 2.041 Å from the (200) face and the lattice parameter in the blue box was calculated as 4.082 Å, which is quite similar to the 4.09 Å for pure silver [15]. In comparison, the distance between atoms was 2.325 Å from the (111) face in the orange box and the lattice parameter was calculated as 4.027 Å, which is smaller than the 4.082 Å obtained outside the nano-sized twins. The smaller lattice parameters in the nano-sized twins confirmed the existence of the shear stress in the electrodeposition process, and the shear stress value could be as high as 65 MPa according to density functional theory (DFT) calculation for generating the nanocrystalline twinning [46].

No nano-sized twins were observed within the Ag-Bi coating, as shown in the high-magnification TEM images in Figure 6a,b. The local SAD pattern images in Figure 6a from the highlighted dark area show the face centered cubic (FCC) structure from the [001] direction, which demonstrated the Ag matrix. However, some random spots were observed, and these spots should be caused by the Bi atoms embedded in the Ag matrix. In the HRTEM image, as shown in Figure 6c, the dark spot without clear atomic structure indicates a possible nanoparticle formed in the electrodeposition process. The atom distance from the (111) face was averaged as 2.397 Å in the orange box, and the lattice parameter was calculated as 4.152 Å. The atom space from the (111) face was measured as 2.336 Å in the highlighted blue box, and the lattice parameter was calculated as 4.045 Å.

### 3.2. Nanoindentation and Inverse Calculation with Three-Dimensional FEA

For the purpose of comparison, the thin Ag-Bi coating was assumed to be transversely isotropic, similar to the Ag coating [27], with the isotropic mechanical property in the plane parallel to the surface. Table 2 summarizes the constitutive relationship and parameters used to describe the anisotropic mechanical properties of Ag-Bi coating. 

**Figure 7:**
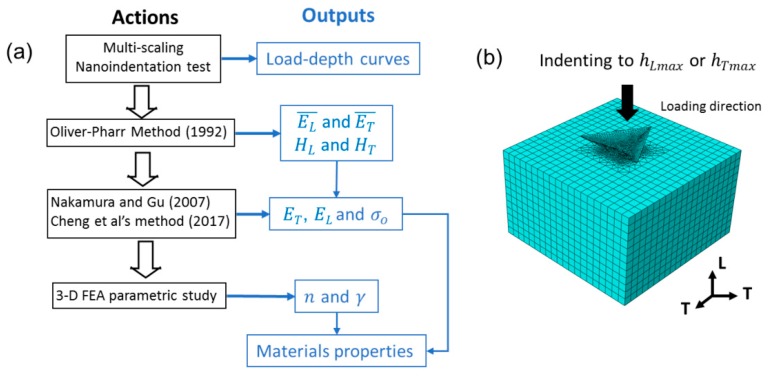
(**a**) The algorithm of inverse calculation and (**b**) the sketch of the FEA model with a Berkovich indenter.

Detailed descriptions of the constitutive relationship can be found in our previous study [27]. To estimate the mechanical properties of Ag-Bi, i.e., the elastic modulus and flow stress from the load-depth curves of the *T* and *L* directions, a three-step method proposed recently was utilized, as shown in Figure 7a. The elastic modulus values of two directions were assumed as the value obtained from the Oliver-Pharr method [47]. The reference strength was determined with empirical equations. Thus, n and γ were determined using 3-D finite element indentation modeling as sketched in Figure 7b.

(1) With the Oliver-Pharr (OP) method [47], the nominal elastic modulus was calculated as $\bar{E_{T}}$ = 94.29±3.82 GPa and $\bar{E_{L}}=87.83\pm 7.52\;\mathrm{GPa}$ for the two directions, respectively. The hardness values in the two directions were calculated as $H_{L}$ = 2.66±0.04 GPa and $H_{T}=2.71\pm 0.07\;\mathrm{GPa}$.
(2) Since the difference between $\bar{E_{T}}$ and $\bar{E_{L}}$ is ~5%, which is quite similar to the previous observation of the Ag coating, $E_{T}$ and $E_{L}$ were replaced with $\bar{E_{T}}$ and $\bar{E_{L}}$ respectively. Meanwhile, with the previous yield strength-hardness relationship at 5 mN [48], $\bar{\sigma_{To}}$ and $\bar{\sigma_{Lo}}$ were calculated as 480 and 410 MPa, respectively. Regarding that the actual yield strength in the two directions ($\sigma_{To}$ + $\sigma_{Lo}$) could be approximated by the sum of the nominal yield strength ($\bar{\sigma_{To}}$ + $\bar{\sigma_{Lo}}$) [25,27], $\sigma_{o}$ was calculated as 445 MPa.
(3) Finally, a 3-D finite element indentation model was developed within the commercial finite element package, ABAQUS, to simulate the indentation process of the Ag-Bi coating in order to inversely determine n and γ. A parametric FEA-based indentation study was carried out to calculate the maximum indentation loads in the two directions to compare with the experimental measurements as target values. As mentioned earlier, the indentation depth was ~300 nm, and the strong indentation size effect (ISE) could be expected considering the thin nature of the Ag-Bi coating. Thus, we first removed the ISE based on the characteristic length ($h^{*}$ = 450 nm [49,50]) for the electrodeposited Ag-Bi coating. It should be noted that the target load values for the simulation are $P_{Tmax}^{S}$ or $P_{Lmax}^{S}$ rather than 5 mN [51]. With one set of n and γ values, the Berkovich indenter penetrates the Ag-Bi coating to the same depth as $h_{Lmax}$, and the corresponding $P_{Lmax}^{FEA}$ in the *L* direction was calculated. Next, the input materials properties were rotated by 90° around the *T* direction, the indenter penetrated the material to $h_{Tmax}$, and the corresponding $P_{Tmax}^{FEA}$ was calculated in the *T* direction. Thus, different sets of $P_{Lmax}^{FEA}$ vs. $P_{Tmax}^{FEA}$ values can be obtained with corresponding n and γ values. The most plausible combination of γ and n can be determined by choosing the corresponding $P_{Lmax}^{FEA}\;\mathrm{and}\;P_{Tmax}^{FEA}$ set closest to the target experimentally measured the data point.

**Figure 8:**
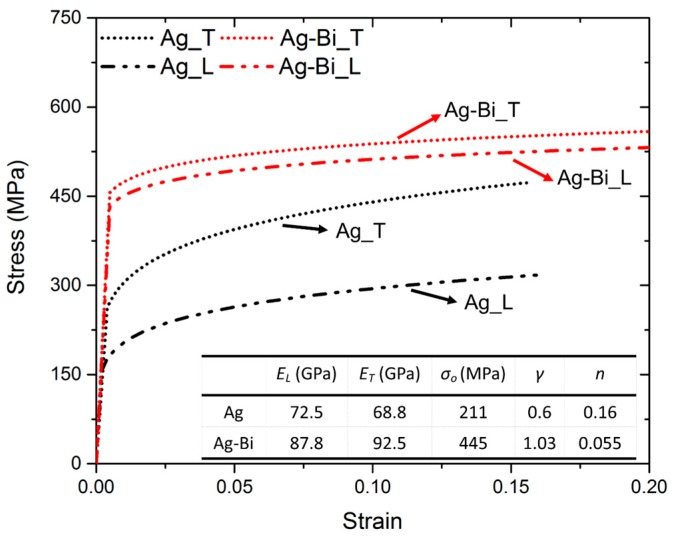
The inverse calculated flow stress for Ag and Ag-Bi in *L* and *T* directions (*Ag_T*, *Ag_L*, *Ag-Bi_T*, and *Ag-Bi_L* denote the flow stress of Ag coating in the *T* direction, Ag coating in the *L* direction, Ag-Bi coating in the *T* direction, and Ag-Bi coating in the *L* direction, respectively).

The estimated average properties of the Ag-Bi coating are: $E_{L}=87.83\;\mathrm{GPa}$, $E_{T}=\;92.46\;\mathrm{GPa},\;\sigma_{o}$ = 445 MPa, γ=1.03, and n=0.055. The obtained flow stress of the two coatings in the two directions are listed and plotted in Figure 8. 

### 3.3. Electrical Resistivity of Ag and Ag-Bi Coatings

The standard electrical resistivity and electrical conductivity of Ag coating are $1.78\pm 0.02\times 10^{-8}\;\Omega \cdot m$ and 97.1 ± 1.2 (%IACS, International Annealed Copper Standard), respectively. As expected, the addition of Bi to the Ag caused the increase of electrical resistivity, and the electrical resistivity of Ag-Bi coating was measured as $1.88\pm 0.02\;\times 10^{-8}\Omega \cdot m$, which indicates a 6% increase of the electrical resistivity. 

## 4. Discussion

### 4.1. Microstructure of the Ag and Ag-Bi Coatings

The main microstructural features to describe a coating include (1) grain size and grain morphology, (2) morphology of grain boundaries and intergranular defects/phases, (3) density of the intergranular defects, and (4) composition distribution across grains and grain boundaries. These features determine the entire elastic-plastic properties of the coatings [52], and the detailed observations are discussed below in conjunction with the inverse results of Ag and Ag-Bi coatings.

#### 4.1.1. Significant Improvement of Flow Stress

A significant improvement on the flow stress was observed for the Ag-Bi coating in both directions: the yield strength of the Ag-Bi coating was improved by 300 MPa (~150%) and 200 MPa (~100%) compared to those of the Ag coating in the *L* direction and *T* direction, respectively. This improvement can be explained by the nanoscale composite microstructures with a large amount of stack faults, i.e., dislocations generated during the electrodeposited process. The TEM images in Figure 5a,b show clean Ag grains with few dislocations for the Ag coating. On the other hand, plenty of internal defects, i.e., dislocations, were observed in the Ag-Bi coating microstructures, as shown in Figure 6a,b. The two images in Figure 6 are quite similar to the TEM images of the mechanically-milled Ag-5.1% Bi alloys as reported by Chithra et al. [11]. The existence of Bi in the Ag matrix cannot be easily distinguished from XRD patterns as compared to other Ag-based alloy systems, i.e., Ag-Al [9], since no new phase was generated. With EDS, the average at% of Bi across the coating thickness would be ~1%. 

In addition, the local HRTEM images illustrate different lattice parameters. The black region in the lower left corner of the orange box can be discerned as the Ag-Bi nanoparticle, which is more difficult to mill in the sample preparation for TEM characterization. Near the nanoparticle in the highlighted orange box as shown in Figure 6c, the lattice parameter was calculated as 4.152 Å, which is much higher than the 4.082 Å [15]. This result indicates that the addition of Bi atoms in the electrodeposited process can expand the Ag lattice, which has been observed in the Ag-5.1% Bi alloys prepared by using high-energy mechanical alloying [11]. Since the possible lattice shrink would also occur, as shown in highlighted blue box of Figure 6c, the introduction of Bi would lead to the variations of the Ag lattice in the current electrodeposited Ag-Bi coating. Meanwhile, within certain regions of the Ag-Bi coating, the lattice parameter was calculated as 4.082 Å, which indicates that there are no solid-solution Ag-Bi nanoparticles in the adjacent regions. Hence, a composite of Ag and Ag-Bi nanostructures was produced with the current electrodeposition process. Those dark spots can act as barriers or obstacles for dislocations to overcome under plastic deformation, and the associated strengthening mechanism resulted from dislocation-particle interaction has been well studied using discrete dislocation dynamics (DD) simulations for nanoscale metallic (NMM) composites [53,54]. Hence, the internal defects and the stiff nanoparticles serves as the main reasons for the improvement of flow stress in Ag-Bi coating.

Additionally, the grain highlighted in Figure 4a for the Ag coating is slightly larger (~181 nm) than the highlighted grain of Ag-Bi coating (~131 nm). This difference indicates that the addition of Bi would also lead to a smaller grain size. The smaller crystalline size would lead to the higher yield strength according to the Hall-Petch relationship [55,56,57]. Meanwhile, it is worth pointing out that there is no intermetallic compound in the current electrodeposited process and we did not observe bright spots from the high-angle annular dark-field (HAAFD) TEM tomography as previously prepared Ni-Bi coatings [14]. 

#### 4.1.2. Significant Decrease of the Hardening Exponent (*n*)

The hardening exponents of two coatings are also determined by the internal microstructure-level features. As the results listed in Figure 8, the hardening exponent of Ag would be as high as 0.16 regarding the larger grains and clean internal structure, compared to those of the Ag-Bi coating. The deformation mechanism of nanocrystalline FCC metals has been well studied and discussed in the previous literature [18,30,45,58]. The twin-containing microstructure in the Ag coating has sufficient space for the storage of dislocations regarding the much fewer nano-sized twins within the current Ag coating in contrast to high-density nano-sized twins a Cu coating prepared by You et al. [30]. The dislocation intersection actions and twin boundaries resulted in the formation of locks in which dislocation trapping and absorption along twin boundaries occurred [18,30,58]. Meanwhile, the rearrangement and annihilation of dislocations led to a low dynamic recovery rate in comparison with that of ordinary grain boundaries [30]. Thus, the high hardening exponent was achieved. The addition of Bi reduced the hardening exponent from 0.16 to 0.055 according to the inverse calculation. Compared to the Ag coating, the Ag-Bi coating has already carried plenty of defects. Therefore, limited spaces for newly-generated dislocations led to a much lower hardening exponent. The high hardening rate, the high uniform elongation, and the high total elongation have been obtained for electrodeposited nanocrystalline materials with thicknesses around 500 μm [30,58]. However, similar behaviors might not be obtained for the current coating with thicknesses less than 10 μm in the FS tensile tests [29] considering inhomogeneous properties [59,60]. 

#### 4.1.3. Slight Improvement of Elastic Modulus

Compared to the Ag coating, an improvement of the elastic modulus was observed for the Ag-Bi coating by 15.3 GPa (~21%) and 23.7 GPa (~34%) in the *L* and *T* directions, respectively. Additionally, the obtained values are higher than the calculated value of 83.8 GPa for the <111> orientation of pure Ag in the previous study [29]. Two factors can lead to the improvement of the elastic modulus: the newly-formed Ag-Bi solid solution and the smaller grain size. Generally speaking, the solid solution can increase the elastic modulus, e.g., more carbon within iron matrix will generate martensite with higher elastic modulus than pure iron [61]. The smaller grain size within Ag-Bi can increase the lattice parameter as shown in Figure 6c, leading to a higher elastic modulus [11]. The inversely calculated elastic modulus of Ag-Bi are consistently higher than those of the Ag coating in two directions. Further studies using DFT will be helpful to support our current results.

#### 4.1.4. Significant Reduced Plastic Anisotropy (*γ*)

**Figure 9:**
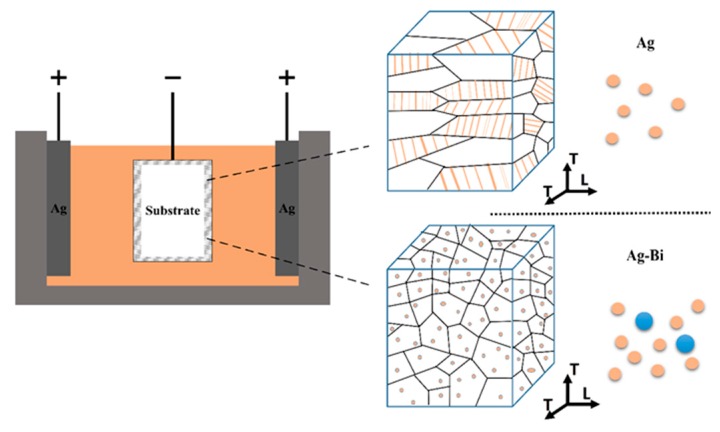
The sketch of Bi addition on the microstructure of the nanocrystalline Ag coating in the the electrodeposition process.

Generally speaking, the electrodeposition process will generate a columnar structure, as previously reported, for FCC metals. Our previous study on Ag coatings confirmed this structure using a nanoindentation-based approach and determined γ=0.6 for Ag coatings with microstructure information from both the plane view and the cross-section view images [30]. The γ value of the currently prepared Ag-Bi coating was calculated as 1.03, which indicates a more homogenous microstructure as compared to the Ag coating. A schematic sketch is shown in Figure 9 to illustrate the effect of a small amount of Bi addition on the structure of the electrodeposited Ag coating: without Bi addition, a columnar structure with larger grains, including nano-sized twins, was formed; with Bi addition, the coating was turned to a finer homogeneous microstructure with Ag-Bi nanoparticles in the Ag matrix.

Since the current Ag and Ag-Bi coatings were prepared with grain size below 1 μm, it would be difficult to directly observe the microstructure by SEM. Hence, further studies should be conducted using TEM and HRTEM to obtain the local microstructural details of the Ag and Ag-Bi coatings in the cross-section view to illustrate the effect of small Bi additions to the grain morphologies.

So far, the effects of Bi addition on the mechanical properties of Ag on the flow stress, hardening exponent, elastic modulus, and the plastic anisotropy are quantitatively obtained. The mechanism behind these effects is explained with the micro- and nanostructures obtained under TEM and HRTEM. Multiple factors, i.e., coating thickness, hardness, and the surface roughness, will determine the final wear performances. Hence, the tribology/wear resistance of coatings is generally characterized by the friction coefficient, volume/weight loss, and track morphology. Among them, volume loss is a characteristic value affected by the mechanical responses from both *L* and *T* directions. According to Archard’s law, volume loss during sliding wear is inversely proportional to the hardness of the coating regardless of different coating compositions, electrodeposited processing parameters, micro/nanostructures, as well as the different contact mechanisms between an abrasive ball and the alloys [62]. The relative reduction of volume loss for the Ag-Bi coating compared to that of Ag coating can then be deduced based on the hardness improvement: with the addition of Bi, a 60% hardness increase is observed from Ag-Bi to Ag coating (~2.67 GPa for Ag-Bi versus ~1.65 GPa for Ag), so a 40% lower volume loss can be expected. Hence, a small amount of Bi addition can greatly improve the strength and the wear resistance of Ag coatings.

### 4.2. Effects of Bi Addition on the Electrical Resistivity

**Figure 10:**
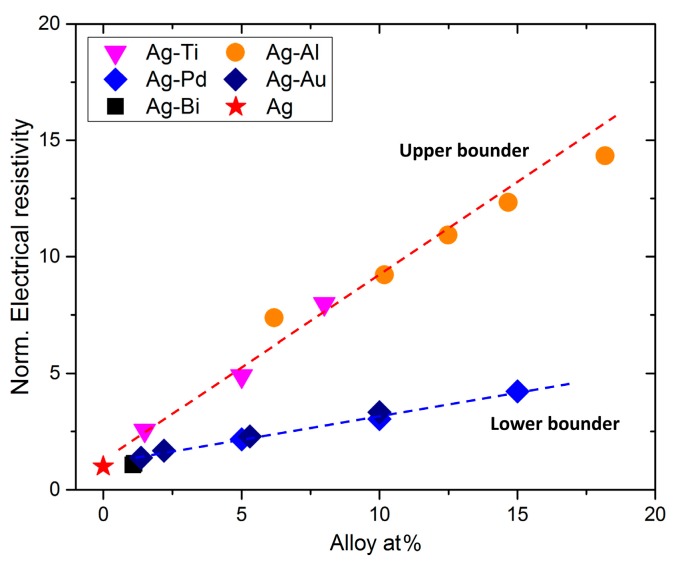
The relationships of alloy at% in Ag matrix versus normalized (Norm.) electrical resistivity ($\frac{\Omega \cdot m\;\left(\mathrm{Ag}-X\right)}{\Omega \cdot m\;\left(\mathrm{Ag}\right)}$) of Ag-Bi, Ag-Al [9], Ag-Pd [10], Ag-Ti [12], and Ag-Au [13] alloys.

As expected, the addition of Bi to Ag matrix increased the electrical resistivity. The electrical resistivity of Ag and Ag-Bi coatings were measured as $1.78\pm 0.02\;\times 10^{-8}\;\Omega \cdot m$ and $1.88\pm 0.02\;\times 10^{-8}\;\Omega \cdot m$, respectively. The ~6% increase in the electrical resistivity is quite small compared to the 60% increase in the hardness. The low electrical resistivity and the high hardness of Ag-Bi coating indicate that the current Ag-Bi nanocomposite is a good candidate for electrical contacting materials. In addition to Ag-Bi alloy, four other Ag alloys were prepared before, and the electrical resistivity of these alloys were measured [1,9,10,13]. The relationship between normalized electrical resistivity at room temperature (the electrical resistivity of Ag alloys, Ω·m (Ag−X), over the electrical resistivity of pure Ag, Ω·m (Ag) and alloy at% is shown in Figure 10. 

In spite of different preparation processing, grain/crystalline size, thermal-mechanical treatment, and geometry shape/size (i.e., bulk, coating, or thin films), a general relationship was found that the higher at% of alloying elements, the higher the electrical resistivity. This relationship is very consistent in the binary alloy system of Ag when the at% of alloying elements is no more than 20%. Meanwhile, an upper bound from Ag-Al and Ag-Ti systems and a lower bound from Ag-Pd and Ag-Au systems are denoted as the green and red dashed curves in Figure 10, respectively. The different phenomena could be attributed to the different atom radii between Ag and the alloy elements. The differences between atom radii in the four alloys are 25.0%, 16.9%, 3.5%, and 5.2% for Ag-Ti (172 pm vs. 215 pm), Ag-Al (172 pm vs. 143 pm), Ag-Au (172 pm vs. 166 pm), and Ag-Pd (172 pm vs. 163 pm), respectively. The total electrical resistivity of one material at room temperature can be attributed to different factors including structural defects (dislocations, vacancies, alloying elements, and impurities) and geometrical scattering (internal and external interfaces) [21]. In the current Ag binary alloys, the alloying elements (atom radii) and the at% of alloying elements within the Ag matrix are two dominant factors determining the electrical resistivity. Although the difference between the atom radii between Bi (230 pm) and Ag (172 pm) is significant, the amount of Bi within the Ag is small (~1%). The increase of electrical resistivity of Ag-Bi is rather small, and the black square in Figure 10 is quite close to the lower bound.

## 5. Conclusions

In this study, the effects of Bi addition on the microstructures, mechanical performances, and electrical resistivity of an electrodeposited Ag coating were investigated. Using nanoindentation in two directions and the inverse calculation, we found that the Ag-Bi coating was much stronger than Ag with a significantly higher yield strength and flow stress. Meanwhile, the anisotropy of the Ag-Bi was significantly reduced as compared to that of the Ag coating, which indicates a more homogenous microstructure. The TEM images demonstrate that the improvement in the mechanical response was originated from the new Ag-Bi solid solution nanoparticles and more defects were generated during the electrodepositing process as compared to the clean structures of Ag, including nano-sized twins. Additionally, the HRTEM results confirm the difference from the lattice parameters of Ag-Bi and nanocrystalline Ag. Since the current Ag-Bi coating presents a higher hardness and low electrical resistivity, this promising method will provide a good solution for electrical contact applications. The methodology used in this study can guide the selection of processing parameters of electrodeposition in optimizing the mechanical properties of nanocrystalline coatings for intended applications.

## Figures and Tables

**Table 1:** Solution composition and operating parameters of electrodeposited Ag and Ag-Bi coatings.

Bath Composition and Plating Parameters	Quantity
**Ag Deposition**	
Silver metal	30 g/L
KCN free	120 g/L
KOH	10 g/L
Temperature	30 °C
Current density	10 mA/cm^2^
Agitation speed	200 rpm
Plating time	30 min
**Ag-Bi Deposition**	
Silver metal	30 g/L
KCN free	120 g/L
KOH	10.35 g/L
Bi(NO_3_)_3_·5H_2_O	0.2425 g/L
Tartaric acid	0.075 g/L
Temperature	30 °C
Current density	10 mA/cm^2^
Agitation speed	200 rpm
Plating time	30 min

**Table 2:** Definitions of the terms and the summary of the constitutive equations used for the inverse calculation.

**(a) Symbol Descriptions**	
$H_{L}$ and $H_{T}$	Hardness in the *L* and *T* directions
$\bar{E_{L}}$ and $\bar{E_{T}}$	Nominal elastic modulus in the *L* and *T* directions
$P_{Lmax}$ and $P_{Tmax}$	Maximum indentation load in the *L* and *T* directions
$h_{Lmax}$ and $h_{Tmax}$	Maximum indentation depth in the *L* and *T* directions
$h^{*}$	Indentation size effect (ISE) characteristic length
γ	The ratio of yield strength
$P_{Lmax}^{S}$ and $P_{Tmax}^{S}$	Maximum indentation load with modification in the *L* and *T* directions
$P_{Lmax}^{FEA}$ and $P_{Tmax}^{FEA}$	Maximum indentation load from FEA in the *L* and *T* directions
$G_{L}$ and $G_{T}$	Shear modulus in the *L* and *T* directions
$\sigma_{o}$	Reference strength
$\epsilon_{L}$ and $\epsilon_{T}$	Isotropic plastic strain in the *L* and *T* directions
*n*	Strain hardening exponent
$\bar{\sigma_{Lo}}$ and $\bar{\sigma_{To}}$	Nominal yield strength in the *L* and *T* directions
$n_{Lo}^{\prime }$ and $n_{To}^{\prime }$	Nominal strain hardening exponent in the *L* and *T* directions
**(b) Constitutive equations**	
**Elastic**	**Plastic**
[εxxεyyεzzγyzγzxγxy]=[1/ET−vLTEL−vTET−vTLEL1/EL−vTLET−vTET−vLTET1/ET1/GL1/GL1/GT] [σxxσyyσzzσyzσzxσxy] $\frac{E_{L}}{E_{T}}=\frac{\nu_{LT}}{\nu_{TL}}\;G_{T}=\frac{E_{T}}{2\left(1+\nu_{T}\right)}\;G_{L}=\frac{E_{T}+E_{L}}{4*\left(1+\left(\frac{\nu_{LT}+\nu_{TL}}{2}\right)\right)}$ $\nu_{T}=0.3\;\nu_{LT}+\nu_{TL}=2\;\nu_{T}$	$\sigma_{L}=\sigma_{Lo}{\left(1+\left(\frac{E_{L}\epsilon_{L}}{\sigma_{Lo}}\right)\right)}^{n}$ for $\sigma_{L}>\sigma_{Lo}$ $\sigma_{T}=\sigma_{To}{\left(1+\left(\frac{E_{T}\epsilon_{T}}{\sigma_{To}}\right)\right)}^{n}$ for $\sigma_{T}>\sigma_{To}$ $\gamma =\frac{\sigma_{Lo}}{\sigma_{To}}$
	**Assumptions for inverse calculation**
	$E_{T}=\bar{E_{T}}\;\mathrm{and}\;E_{L}=\bar{E_{L}}$ $\sigma_{Lo}+\sigma_{To}=2\sigma_{o}=\sigma_{Lo}^{\prime }$ + $\sigma_{To}^{\prime }$
